# Predictors and Frequency of Conduction Disturbances After Open-Heart Surgery

**Published:** 2008-02-01

**Authors:** Zahra Emkanjoo, Mansour Mirza-Ali, Abollfath Alizadeh, Saied Hosseini, Mohammad Vahid Jorat, Mohammad Hossein Nikoo, Mohammad Ali Sadr-Ameli

**Affiliations:** 1 The department of Pacemaker and Electrophysiology, Rajaie Cardiovascular Research and Medical Center, Tehran, IRAN; 2 The department of Cardiology, Rajaie Cardiovascular Research and Medical Center, Tehran, IRAN; 3 The department of Cardiovascular surgery, Rajaie Cardiovascular Research and Medical Center, Tehran, IRAN

**Keywords:** Post operative conduction disturbances, permanent pacemaker

## Abstract

**Introduction:**

The risk of developing conduction disturbances after coronary bypass grafting (CABG) or valvular surgery has been well established in previous studies, leading to permanent pacemaker implantation in about 2% to 3% of patients, and in 10% of patients undergoing repeat cardiac surgery. We sought to determine the incidence, features and predictors of conduction disorders in the immediate post-operative period of patients subjected to open-heart surgery, and the need for permanent pacemaker implantation.

**Material and Method:**

We prospectively studied 374 consecutive patients who underwent open-heart surgery in our institution: coronary artery bypass (CABG) (n=128), Mitral valve replacement(MVR)(n=18), aortic valve replacement(AVR) (n=21), MVR and AVR(n=56), repair of ventricular septal defect (VSD) (n=51), repair of tetralogy of Fallot (TOF) (n=57),CABG and valvular surgery (n=6), others (n=37).

**Results:**

Among 374 patients included in our study (mean age 34.46±25.68; 146 males), 192 developed new  conduction disorders: symptomatic sinus bradycardia in 8%, atrial fibrillation with slow ventricular response (AF) in 4.5%, first-degree atrioventricular block (AVB)in 6.4%, second-degree AVB in 0.3%, third-degree AVB in 7%, new right bundle branch block (RBBB) in 33%, and new left bundle branch block (LBBB) in 2.1%. In 5.6% patients, a permanent pacemaker was implanted, 47.6% of them underwent valvular surgery. In 44.1% of patients the conduction defects occurred in the first 48 hr. after surgery. In CABG group, 29.7% of patients developed new conduction disturbances; the most common of them was symptomatic sinus bradycardia. After valvular surgery 44.2% of patients developed conduction disturbances, of those the most common was  atrial fibrillation with slow ventricular response . After VSD and TOF repair, the most common conduction disturbance was new RBBB. Perioperative myocardial infarction (MI) occurred in  1.9% of  patients. The occurrence conduction disturbance was compared with patient age, sex, occurrence of perioperative MI, ejection fraction (EF), postoperative use of ß-adernergic receptor blocking agents and digitalis and type of cardiac surgery. By regression analysis there was a correlation between type of surgery and new conduction defects, being significant for CABG and TOF repair. Only the occurrence of perioperative MI was related to PPM implantation.

**Conclusion:**

Irreversible AVB requiring a PPM is an uncommon complication after open-heart surgery. Peri-operative MI is a risk factor.

## Introduction

The risk of developing conduction disturbances after coronary bypass grafting (CABG) or valvular surgery has been well established in previous studies, leading to permanent pacemaker implantation in about 0.4 to 1.1% of patients after isolated CABG and from 3% to 6% after a valvular operation [1].

The conduction defects include partial and complete bundle branch blocks, various degrees of atrioventricular (AV) block and sinus node dysfunction. Right bundle branch block (RBBB) was the most frequently noted abnormality [2].

Conduction disorders requiring PPM after cardiac surgery are explained by one of the following two mechanisms: 1) operative procedures in close proximity to the sinoatrial or AV nodes or the His bundle might result in trauma to the conduction system; or 2) extensive coronary artery disease might compromise myocardial protection during intraoperative cardioplegic arrest, contributing to ischemic injury to the conduction system [3].

A new and persistent conduction disorder after surgery significantly increases the risk of subsequent arrhythmic events [4,5]. A significant correlation between recorded ventricular arrhythmias and conduction defects has been reported in patients undergoing surgery for congenital heart disease [6,7]. Therefore a careful investigation and treatment approach should be considered in these patients soon after surgery.

The aim of this study was to evaluate the type and frequency of conduction disturbances as well as the frequency of requirement for permanent pacing and related predictors after open heart surgery.

## Materials and Methods

A total of 374 consecutive patients who underwent open-heart surgery between 2005 and 2006 were included in this prospective study. All procedures were performed under moderate systemic hypothermia with cold crystalloid anterograde cardioplegia (MARTINDALE) repeated every 15 minutes for myocardial protection.

We excluded from this study patients with conduction disturbances (LBBB, RBBB, LAHB, first degree AVB, second degree AVB and third degree AVB) before surgery.

To assess conduction disturbances, a 12-lead ECG was recorded routinely 24 hours before surgery, 5 hours after surgery, and for the first 4 postoperative days. In addition, ECG was performed more frequently as required or daily if conduction disturbances were present, up to hospital discharge. Continuous monitoring was routinely performed in all patients in intensive care unit and continued thereafter if conduction disturbances or pacing from epicardial electrodes were present. Measurement of total CK and CK-MB were made every 8 hours for the first 24-36 hours if perioperative MI was suspected. The diagnosis of MI after cardiac surgery was made by finding of new, persistent Q waves, elevated levels of myocardial enzymes and new regional wall motion abnormality on echocardiography. The study protocol was approved by the institutional review board.

## Statistical analysis

Statistical analysis was performed using the statistical software SPSS 13 for Windows. For univariate analysis, Fisher's exact test and Chi-square test were used to compare categorical variables between groups. Logistic regression analysis was used for continuous variables. Predictors of conduction disturbances were identified using univariate analysis with a p<0.2 and were included as independent variable in a stepwise logistic regression analysis. A p value of <0.05 (two-tailed) was considered statistically significant.

## Results

A total of 374 patients consented to participate in this study. Mean age was 34.46±25.68 (median 40), and 188 of them (50.3%) were women. Echocardiographic data before surgery revealed left ventricular ejection fraction (LVEF) > 35% in 344 (91.9%) patients. After surgery 340 (90.9%) patients had  LVEF>35%.

Table 1 shows the baseline characteristics of patients and the preoperative drugs used during the month preceding operation. Therapy with AV nodal blockers was discontinued after development of postoperative second degree and third degree AVB.

The types of  cardiac surgery were as follows: 1) Coronary artery bypass graft (CABG) (n=128,34.2%); 2) Mitral valve replacement (MVR) and aortic valve replacement (AVR) (n=56,15%); 3) AVR (n=21,5.6%); 4) MVR (n=18,4.8%); 5) Ventricular septal defect (VSD) repair (n=51,13.6%); 6) Repair of tetralogy of Fallot (TOF) (n=57,15.2%); 7) CABG and valvular surgery(n=6,1.6%); and others (n=37,9.89%). Perioperative MI occurred in  7 patients (1.9%).

### Postoperative conduction disturbances

Postoperative conduction disturbances occurred in 192 patients (Table 2): symptomatic sinus bradycardia in 30 patients (8%), atrial fibrillation with slow ventricular response (AF) in 17 patients (4.5%), first-degree atrioventricular block (AVB) in 24 patients (6.4%), second-degree AVB in 1 patient (0.3%), third-degree AVB in 26 patients (7%), new RBBB in 86 patients (23%), and new LBBB in 8 patients (2.1%). In 165 of patients the conduction defects occurred in the first 48 hours after surgery.

Postoperative AF was transient in all cases. Sinus bradycardia was still present in 4 patients up to discharge. First degree AVB was present in 17 patients, second degree AVB in 1 patient, third degree AVB in 19 patients at the time of discharge. RBBB was present in 69 patients and LBBB in 7 patients.

### Postoperative conduction disturbances according to type of operation

In CABG group, 38 patients developed new conduction disturbances; the most common of them was symptomatic sinus bradycardia. After valvular surgery 42 patients developed conduction disturbances, of those the most common was atrial fibrillation with slow ventricular response. After VSD and TOF repair, 40 and 55 patients developed new conduction defects, respectively; the most common conduction disturbance was new RBBB. In CABG and valvular surgery group, 4 patients had new conduction defects; the most common was sinus bradycardia. (Table 3)

### Consequences of conduction disturbances

In 21 (5.6%) patients, a PPM was implanted, 47.6% of them had undergone valvular surgery. Nineteen patients required PPM implantation due to complete AVB and 2 patients due to symptomatic sinus bradycardia.

In patients with congenital heart disease 6.29% needed PPM implantation. In valvular surgery group, 8.42% and in CABG group 1.56% of patients required a PPM. We followed the patients who required PPM implantation for up to 6 months and found that conduction defects (AVB and sinus tachycardia) were persistent in all of them.

### Multivariate analysis

We assessed the effect of age, sex, perioperative myocardial infarction, ejection fraction (EF), postoperative ß-adernergic receptor blocking agents, use of digitalis, and underlying heart disease on the occurrence of new conduction disturbances. Postoperative use of beta-blockers and digoxin as well as LVEF was not found to be associated with occurrence of conduction defects.

We found a correlation between underlying heart disease with persistent new conduction defects, being significant for valvular heart disease and congenital heart disease (p value<0.001, <0.001, respectively).

Logistic regression analysis showed that type of conduction defects which occurred immediately after surgery (sinus bradycardia, first degree AVB, third degree AVB, RBBB and LBBB) were predictors of persistence of conduction defects up to discharge. Only the occurrence of perioperative MI was related to PPM implantation. (Table 4)

## Discussion

Conduction disturbances may occurr after cardiac operations and may require cardiac pacing. Sinus node dysfunction, bundle branch blocks of variable degree, and completed AV block may reverse during the hospital stay. The requirement of PPM after surgery is a rare but serious complication.

In one study [1], 2.1% of patients who underwent open-heart surgery, required permanent cardiac pacing postoperatively. The most important predictive factors were: 1) preoperative evidence of conduction disorder; 2) advanced age; 3) valvular surgery, especially tricuspid valve surgery; and 4) poor myocardial protection.

Tuzcu, et al. reported that 5.5% of their patients, who underwent CABG, developed a new intraventricular conduction defect that persisted to hospital discharge. RBBB occurred in 85% of them, LBBB in 4% and nonspecific intraventricular conduction defect in 11%. But heart block was not reported in their series [8].

Meimoun P, et al. examined the incidence, predictors, and evolution of postoperative AVB after mitral valve repair. They showed that the preoperative variables were not related to postoperative AVB. A lesser systemic hypothermia during surgery was the only, modestly independent predictor of postoperative AVB. PPM implantation was required in 2.6% of their patients and all had persistent third degree AVB [9].

Erdogan HB, et al. showed that irreversible AVB requiring PPM implantation is an uncommon complication after AVR (4.1%). Risk factors were annular calcification, bicuspid aortic valve, female sex, presence of RBBB or LBBB, prolonged total perfusion time, and hypertension [10].

Cook DJ, et al. hypothesized that population aging and increased use of beta-blockers would increase the incidence of new conduction defects after coronary surgery. Their results were the opposite of those predicted. They identified a changing incidence, type, and natural history of conduction defects after CABG. They demonstrated a decrease in the incidence of new conduction defects (19% to 6%), as well as a qualitative change in the defects identified, from a RBBB to first degree AVB. They noted age, number of vessels bypassed, type of cardioplegia, year of operation and intraaortic balloon counterpulsation as predictors of postoperative conduction defects [11].

In one study that examined the occurrence of arrhythmia in patients with congenital heart disease, the incidence of complete RBBB and complete RBBB-LAH following VSD repair was 33% and 6.6%, respectively, and for TOF was 55% and 5.2%, respectively. Complete AVB was seen in 2 patients following VSD repair, and none of TOF patients developed complete AVB [12].

Deanfield JE, et al. reported that in patients who underwent correction of TOF, complete RBBB occurred in 95% of patients, RBBB and LAD in 9% and progressive conduction defects, either LAH or RBBB, developed during follow-up in 11% [13]. Okoroma EO et al. reported an incidence of 33% RBBB and complete RBBB following VSD repair and 55% for TOF, respectively [14]. Although cardiac conduction defects are often encountered after cardiac surgery, the incidence of a PPM implantation is not so frequent, and it has been reported to be around 2.2% [15].

In our series the persistent conduction defects occurred in 124 patients (33.2%), the most common of them were RBBB (n=69) followed by third degree AVB (n=19). In CABG group 14.06%, in valvular surgery group 25.26%, in congenital heart disease group 55.94%, and in CABG and valvular surgery group 50% of patients developed persistent conduction defects. A PPM required to be implanted in 5.6% of patients. In patients with congenital heart disease 6.29% needed PPM implantation. In valvular surgery group, 8.42% and in CABG group 1.56% of patients required a PPM.

Our results are comparable with the previous studies and show that the majority of post CABG conduction defects resolve spontaneously before hospital discharge and rarely require a PPM. In our series, in only 1.56% of these patients significant and prolonged bradyarrhythmias developed and required treatment with a PPM. We found a high incidence of perioperative MI in patients who need PPM implantation (p <0.001). We also found a correlation between underlying heart disease with persistent new conduction defects, being significant for valvular heart disease and congenital heart disease (p value<0.001 and <0.001, respectively).

It is worth pointing that the conduction defects which occurred immediately after surgery (sinus bradycardia, first degree AVB, third degree AVB, RBBB and LBBB) were predictors of persistence of conduction defects to discharge. In our study, the incidence of significant conduction defects requiring PPM implantation was high compared to what would be expected from similar studies. It could be explained by poor myocardial protection techniques that led to myocardial and conduction tissue injury during operation in our patients.

## Study limitations

A limitation of this study is that operative variables such as cardiopulmonary bypass time, aortic cross-clamp time and myocardial temperatures were not available; therefore the cooling of His bundle-AV node could not be assessed.

## Figures and Tables

**Table 1:**
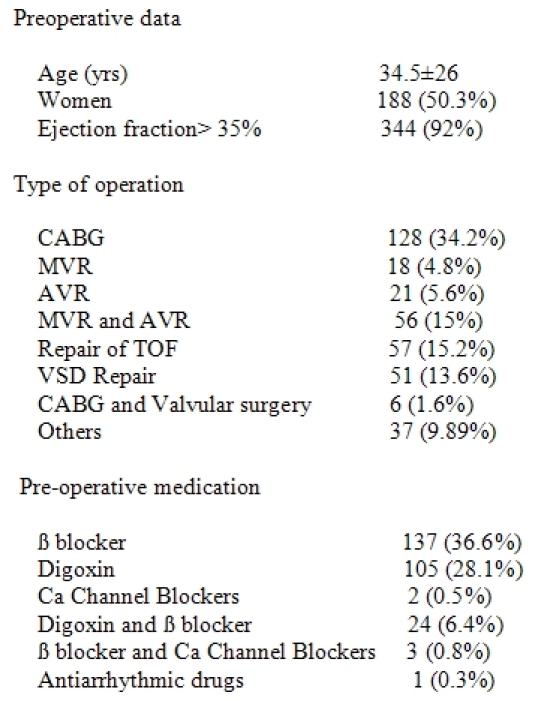
Baseline patient characteristics

TOF= tetralogy of Fallot; VSD= ventricular septal defect; MVR= mitral valve replacement; AVR= aortic valve replacement; CABG= coronary artery bypass graft

**Table 2:**
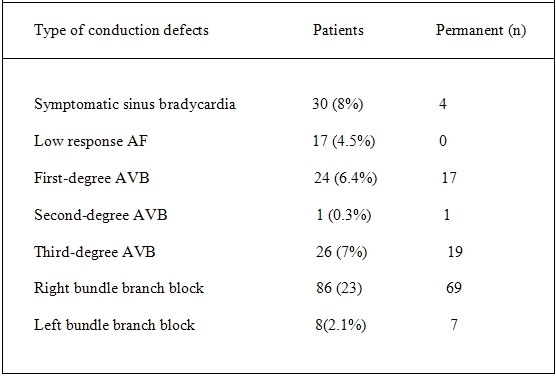
Conduction defects after open-heart surgery

**Table 3:**
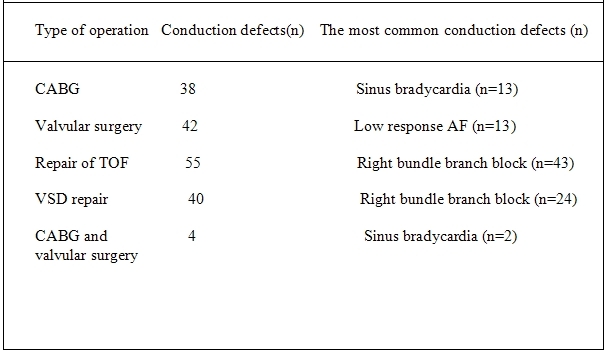
Correlation between type of operation and conduction defects

**Table 4:**
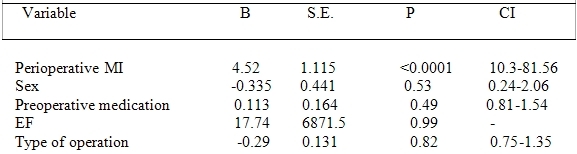
Logistic Regression Analysis of Risk Factors for PPM implantation

## References

[R1] Goldman BS, Hill TJ, Weisel RD (1984). Permanent pacing after open-heart sugery: acquired heart disease. Pacing Clin Electrophysiol.

[R2] Baerman JM, Kirsh MM, Buileir M (1987). Natural history and determinants of conduction defects following coronary artery bypass surgery. Ann thorac Surg.

[R3] Gordon SR, Ivanov BJ, Cohen G (1998). Permanent cardiac pacing after a cardiac operation: Predictoing the use of permanent pacemakers. Ann thorac surg.

[R4] Pires LA, Wagshal AB, Lancey R (1995). Arrhythmias and Conduction Disturbances after Coronary graft Surgery: Epidemiology, management, and prognosis. Am Heart J.

[R5] El-Khally Z, Thibault B, Staniloae C (2004). Prognostic significance of newly acquired bundle branch block after aortic valve replacement. Am J Cardiol.

[R6] Blake RS, Chung EE, Wesley H (1982). Conduction  defects, ventricular arrhythmias, and late death after surgical closure of ventricular septal defect. Br Heart J.

[R7] Makoto N, Tokuko SH, Akihito S (2004). Arrhythmias Late After Repair of Tetralogy of Fallot-A Japanese Multicenter Study. Circ J.

[R8] Tuzcu EM, Emre A, Goormastic M (1990). Incidence and prognostic significance of intrventricular conduction abnormalities after coronary bypass surgery. J Am Coll Cardiol.

[R9] Meimoun P, Zeghdi R, D'Attelis N (2002). Frequency, predictors, and consequences of atrioventricular block during mitral valve repair. Am J Cardiol.

[R10] Erdogan HB, Kayalar N, Ardal H (2006). Risk factors for requirement of permanent pacemaker implantation after aortic valve replacement. J Card Surg.

[R11] Cook DJ, Bailon JM, Douglas TT (2005). Changing incidence, type, and natural history of conduction defects after artery bypass grafting. Ann thorac Surg.

[R12] Niimura I, Shibata T, Haraguchi T (1981). Pre- and postoperative arrhythmias in congenital heart disease: from the results of suyrery using surface- induced deep hypothermia. Jpn Circ J.

[R13] Deanfield JE, McKenna WJ, Hallidie-smith KA (1980). Detection of late arrhythmia and conduction disturbances after correction of teteralogy of Fallot. Br Heart J.

[R14] Okoroma EO, Guller B, Maloney JD (1975). Etiology of right bandle-branch lock pattern after surgical closure of ventricular-septal defects. Am Heart J.

[R15] Lewis JW, Webb CR, Pickard SD (1998). The increased need for a permanent pacemaker after reoperative cardiac surgery. J Thorac Cardiovasc Surg.

